# The Influence of COVID-19 on Patient Mobilization and Injury Attributes in the ICU: A Retrospective Analysis of a Level II Trauma Center

**DOI:** 10.3390/traumacare4010005

**Published:** 2024-02-07

**Authors:** Yelissa Navarro, Elizabeth Huang, Chandler Johnson, Forrest Clark, Samuel Coppola, Suraj Modi, Gordon L. Warren, Jarrod A. Call

**Affiliations:** 1 Medical College of Georgia, AU/UGA Medical Partnership, Athens, GA 30602, USA; 2 Department of Physical Therapy, Georgia State University, Atlanta, GA 30302, USA; 3 Department of Physiology & Pharmacology, University of Georgia, Athens, GA 30602, USA

**Keywords:** early mobilization, medical records, trauma severity

## Abstract

The objectives of this study were to determine the effect of COVID-19 on physical therapy (PT) mobilization of trauma patients and to determine if mobilization affected patient course in the ICU. This retrospective study included patients who were admitted to the ICU of a level II trauma center. The patients were divided into two groups, i.e., those admitted before (*n* = 378) and after (*n* = 499) 1 April 2020 when Georgia’s COVID-19 shelter-in-place order was mandated. The two groups were contrasted on nominal and ratio variables using Chi-square and Student’s *t*-tests. A secondary analysis focused specifically on the after-COVID patients examined the extent to which mobilization (*n* = 328) or lack of mobilization (*n* = 171) influenced ICU outcomes (e.g., mortality, readmission). The two groups were contrasted on nominal and ratio variables using Chi-square and Student’s *t*-tests. The after-COVID patients had higher injury severity as a greater proportion was classified as severely injured (i.e., >15 on Injury Severity Score) compared to the before-COVID patients. After-COVID patients also had a greater cumulative number of comorbidities and experienced greater complications in the ICU. Despite this, there was no difference between patients in receiving a PT consultation or days to mobilization. Within the after-COVID cohort, those who were mobilized were older, had greater Glasgow Coma Scale scores, had longer total hospital days, and had a lesser mortality rate, and a higher proportion were female. Despite shifting patient injury attributes post-COVID-19, a communicable disease, mobilization care remained consistent and effective.

## 1. Introduction

In 2020, due to the coronavirus pandemic caused by SARS-CoV-2 (COVID-19), the United States was forced to enact sudden measures to help alleviate the spread of the virus. A “shelter-in-place” approach was executed, canceling many large gatherings and encouraging individuals to stay at home. Travel was strictly controlled, especially internationally, and a variety of protective public health initiatives were put forward, namely an emphasis on handwashing, mask-wearing, and social distancing with “stay-at-home” orders [1]. Hospitals experienced a nationwide drop in overall admissions beginning in March of 2020, including acutely ill non-COVID-19 patients [2]. It has been theorized in current literature that a large reason for this was the avoidance of patients in seeking hospital care, mainly in response to media messaging and hesitancy to leave home with “shelter-in-place” orders in effect [2,3]. With these restrictions in mind, we sought to assess the pandemic’s effect on intensive care unit (ICU) trauma admissions and care.

In the treatment of critically ill patients, early mobilization and early physical therapy (PT) intervention have been associated with a reduced stay in the ICU [4–13], reduced ICU healthcare costs [5,6,11,14–17], reduced length of stay in the hospital [5,12–14,18–25], and decreased hospital readmissions [4]. Additionally, improved patient functional outcomes and decreased need for care after hospital discharge have been associated with early mobilization [5,7–10,12–14,18–25]. However, PT and mobilization require close human interaction, and after March 2020, these interactions were taking place within the global context of a highly communicable disease. Therefore, the first objective of our study was to determine whether the onset of COVID-19 was associated with a change in the percentage of patients receiving a PT mobilization order and time to mobilization. The second objective of our study was to determine if there were differences in patient characteristics, injury attributes, hospital stay, and readmission rates for patients in the “after COVID onset” group that did or did not receive mobilization in the ICU.

## 2. Methods

This was a retrospective analysis of trauma patients who were admitted to the ICU at Piedmont Athens Regional Medical Center (PARMC) in Athens, Georgia. PARMC is a 360-bed, non-profit hospital that offers a level II trauma center and serves Athens and 17 counties in Georgia. Approval for this study was granted by the Piedmont Institutional Review Board and Piedmont Athens Regional, IRB #: 1751179-1 Piedmont.

**Figure 1.**
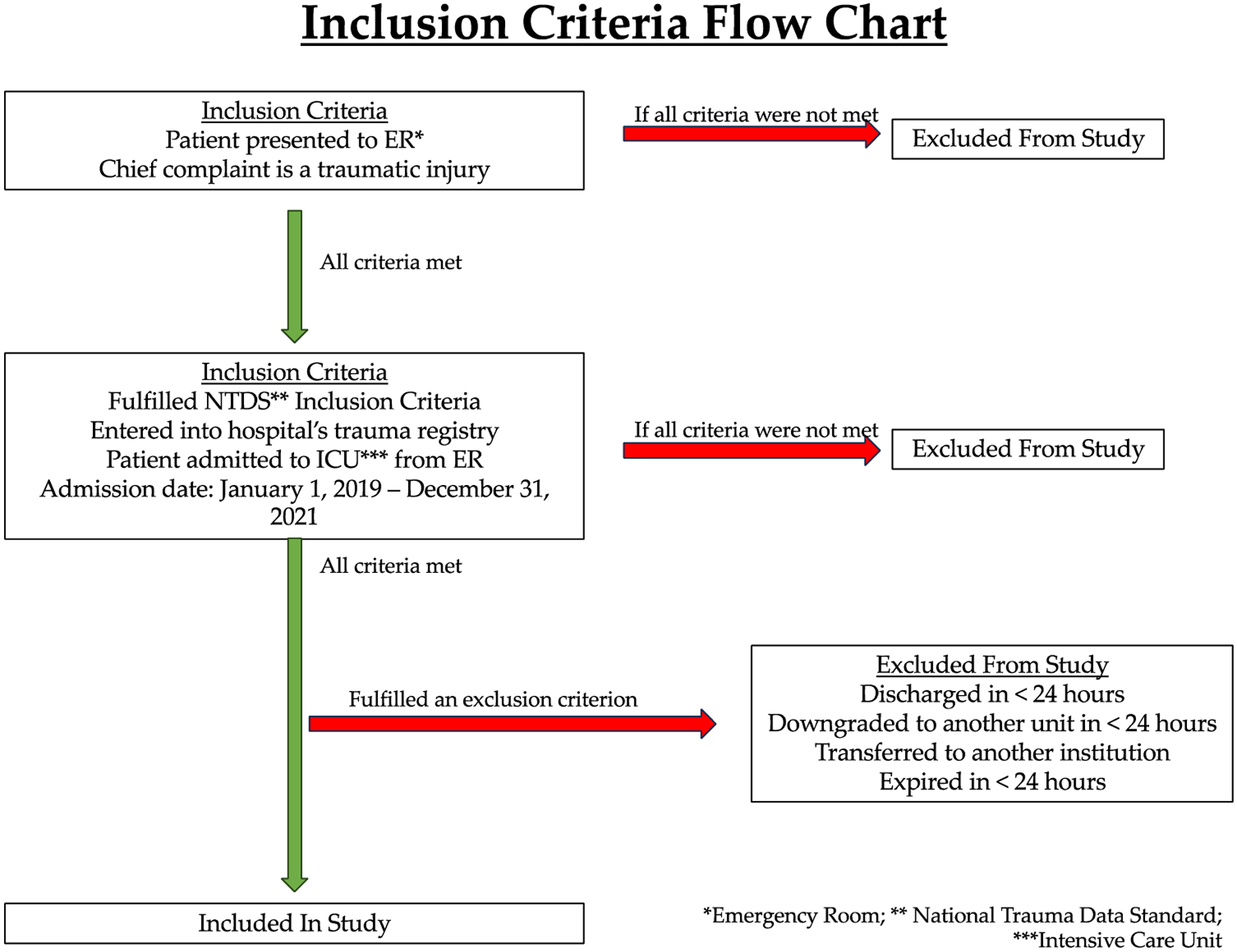
Inclusion criteria of patients in this study.

This study specifically looked at patients admitted to the ICU from the emergency room (ER) at PARMC due to traumatic injury. Our analysis included patients who were admitted from 1 January 2019 to 31 December 2021, and patients were selected using inclusion criteria outlined by the National Trauma Data Standard (NTDS). ER patients were first entered into the trauma registry if they fulfilled NTDS inclusion criteria. Our study then selected patients who were recorded in the trauma registry and were subsequently admitted to the ICU. Patients meeting these criteria were identified in the hospital’s trauma registry, a database that provided detailed information including demographics, mechanisms of injury, and patient outcomes. In total, 877 patients met the criteria for our analysis (Figure 1).

**Figure 2.**
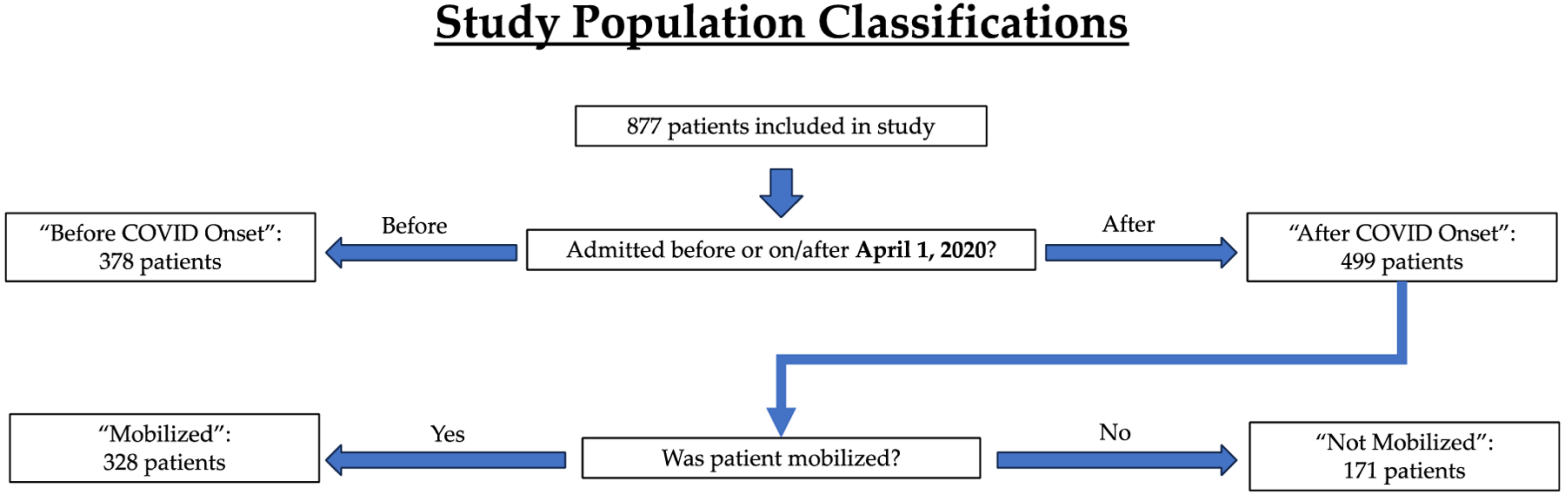
Study population and key parameters identified for patient classification.

The time period for data collection in our study encompassed the COVID-19 pandemic and the shutdowns that followed in its wake. Patients were divided into two groups: “before COVID onset” (*n* = 378) and “after COVID onset” (*n* = 499), meaning after the onset of COVID-19 (Figure 2). To demarcate these two groups, the threshold date for the COVID-19 pandemic was defined as 1 April 2020, with patients admitted to the ICU on or before 31 March 2020 classified as “before COVID onset” and those admitted on or after 1 April 2020 as “after COVID onset”. Any patients who were discharged less than 24 h after admission, downgraded to another unit less than 24 h after ICU admission, transferred to another institution, or who expired less than 24 h after admission were not included in the patient group (Figure 1). Analyses were then performed to look for differences in patient and injury characteristics as well as changes in care, such as PT consultation, in the “before COVID onset” and “after COVID onset” settings. A secondary analysis was eventually conducted to determine patient characteristics and outcomes within the “after COVID onset” group that were mobilized (Mobilized) or were not mobilized (Not Mobilized) (Figure 2).

To analyze differences in patient outcomes, clinical data were extracted from the hospital’s trauma registry and patient charts. The variables extracted consisted of patient demographics, injury types, chief complaints, injury severity scores, hospital course, PT consultation, and time to mobilization. Patient demographics were defined as age, sex (male or female), ethnicity (Hispanic/Latino or non-Hispanic/Latino), and race [White/Caucasian or Other (Black/African American, Asian, Hispanic, American-Indian/Alaskan Native, Pacific Islander, or refused to answer)]. Injury types were defined as blunt or penetrating according to the descriptions of trauma types in the AOTR Alliance of Trauma Registry Resource Manual [26]. Chief mechanisms of injury were categorized as falls, gunshot wounds, motor vehicle crashes, or other (assault, bicycle, burn, knife, pedestrian, puncture wound, other blunt mechanism, other penetrating mechanism, and unknown). Recorded vitals upon Emergency Department (ED) admission were weight, height, and body mass index. Injury severity scores included the Glasgow Coma Score (GCS), Injury Severity Score (ISS), severe ISS (scores greater than 15), New Injury Severity Score (NISS), Trauma and Injury Severity Scores (TRISS), and the Revised Trauma Score (RTS) (see Table 1). Hospital course-of-care data included the day of the week the patient was admitted to the ER, length of stay in the ICU, length of stay in the hospital itself, days spent on the ventilator, and readmission. PT was indicated on the data record sheet if the patient received a PT consultation and was later mobilized. Time to mobilization was recorded as the number of days it took from the day of admission to the first instance of patient mobilization by PT. For readmissions, data that were recorded were the number of days post-ED admission before the patient was readmitted and the number of days patients were readmitted.

A chart review was conducted twice for each patient, each time by an independent researcher. If there was a disagreement between the two researchers on a given patient, a third review of the record was performed by another researcher to reconcile the disagreement. The criteria for designating a patient as being mobilized included supine-to-sit or sit-to-stand transfers, walking, or ascending/descending stairs. The level of assistance provided during locomotion or a transfer was not considered in defining mobilization. Dependent rolling or sliding in bed, moving the bed to a chair position, and transfer to the operating room table or cardiac chair were excluded from our definition.

Other important variables that were extracted included comorbidities, complications, and mortality. Comorbidities recorded from charts were alcohol-use disorder, drug-use disorder, currently receiving chemotherapy, congenital anomalies, congestive heart failure, smoker, chronic renal failure, previous cerebrovascular accident, diabetes, currently has cancer, possesses an advanced directive limiting care, current inability to perform activities of daily living, previous history of angina, previous history of myocardial infarction, peripheral vascular disease, hypertension, chronic obstructive pulmonary disease, steroid use, liver cirrhosis, dementia, attention-deficit/hyperactivity disorder, any other major psychiatric illness not mentioned, and any other disorders not mentioned. Complications recorded were deep surgical infection, previous history of drug/alcohol-use disorder development, deep venous thrombosis, compartment syndrome, graft/prosthesis/flap failure, myocardial infarction, organ space infection, osteomyelitis, pneumonia, pulmonary embolism, sepsis, stroke/cerebrovascular accident, superficial infection, unplanned intubation, unplanned return to the operating room, and urinary tract infection. Mortality was recorded as whether the patient was discharged alive or expired.

Data were extracted in two stages with the goal of determining whether patient mobilization following a traumatic injury was affected by the onset of COVID. In the first stage, all trauma patients who were admitted to the ER and fulfilled the NTDS criteria were recorded on the PARMC trauma registry. This trauma registry included mechanisms of injury, injury code, hospital stay duration, where the patient was discharged afterward, and the ICD-10 diagnosis of, at maximum, six of the patients’ injuries. In the second stage, patient medical records were analyzed for all patients on the trauma registry who were then admitted to the ICU. During this chart review, each patient record was analyzed by two independent investigators to extract and confirm the variables listed above and to confirm the previously extracted data from the trauma registry.

All patients were de-identified during the statistical analysis phase. The two groups (i.e., “before COVID onset” and “after COVID onset”) were compared using Mann–Whitney tests because the datasets were not normally distributed. Results are reported as median and interquartile range. The two groups were contrasted on nominal variables using Chi-square tests. All statistical analyses were performed using JMP statistical software (JMP 16, SAS, Cary, NC, USA).

## 3. Results

### 3.1. “Before and after COVID Onset” Patient Characteristics, Injury Attributes, and Mobilization

For the 877 patients, demographic, basic clinical measures, and patient injury types that were ratio data are summarized in Table 2. Median patient age was not different between the two groups (i.e., the “before COVID onset” and “after COVID onset” groups) (50 vs. 55 years, *p* = 0.70). There was a significant race distribution difference such that Other (Asian, Black/African American, Native Hawaiian, Pacific Islander) represented a greater proportion of patients in the “after COVID onset” group vs. the “before COVID onset” group (26% vs. 19%, *p* = 0.016). There were no statistically significant differences in basic clinical measures (e.g., height, weight, body mass index) between groups (Table 2).

**Figure 3.**
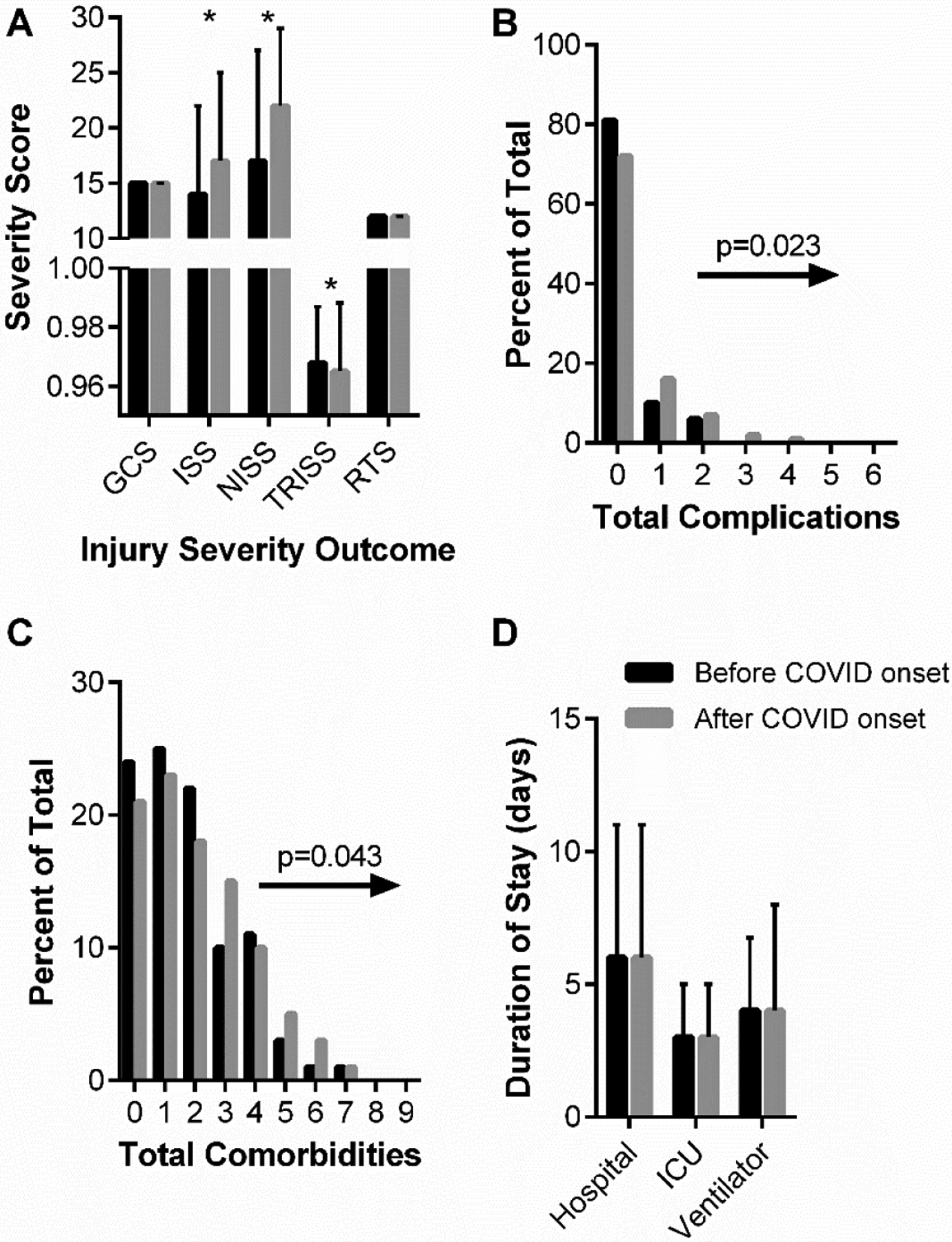
“before and after COVID onset” patient injury severity (**A**), cumulative complications (**B**), cumulative comorbidities (**C**), and hospital durations (**D**). (**A**) Glasgow Coma Score (GCS), Injury Severity Score (ISS), New Injury Severity Score (NISS), Trauma Score and Injury Severity Score (TRISS), and Revised Trauma Score (RTS) are shown as median and interquartile range. Data analyzed using Mann–Whitney test with * representing statistical difference between groups (*p* < 0.05). (**B**) Chi-squared analysis of distribution shifts of total patient complications between “before COVID onset” and “after COVID onset” cohorts. Statistical *p*-value and distribution shift direction are indicated. (**C**) Chi-squared analysis of distribution shifts of total patient comorbidities between “before COVID onset” and “after COVID onset” cohorts. Statistical *p*-value and distribution shift direction are indicated. (**D**) Hospital, ICU, and ventilator durations are shown as median and interquartile range. Data analyzed using Mann–Whitney test.

Blunt trauma was the most common injury type, independent of group, and this was reflected within the chief complaint data as high proportions for complaints identified as falls (38% of total) and motor vehicle accidents (49% of total). There was a statistically significant chief compliant distribution shift such that gunshot wounds represented a greater proportion of complaints in the “after COVID onset” group vs. the “before COVID onset” group (8.2% vs. 4.5%, *p* = 0.046). Overall, a higher proportion of “after COVID onset” patients were classified as severely injured, i.e., an ISS greater than 15, compared to “before COVID onset” patients (58.5% vs. 46.8%, *p* < 0.001). In addition, ISS and NISS scores were, on average, significantly higher in the “after COVID onset” patients (Figure 3A). TRISS scores, which estimate the survivability of patients, were significantly lower in the “after COVID onset” patients (Figure 3A). Finally, “after COVID onset” patients presented to the ICU with greater total comorbidities and experienced greater total complications while in the ICU (Figure 3B,C).

Individual comorbidities and complications for the before and “after COVID onset” analysis are reported in Tables 3 and 4, respectively. A greater percentage of “after COVID onset” patients presented to the ICU with disseminated cancer (6% vs. <1%, *p* < 0.001) and advance directives limiting care (~13% vs. 2%, *p* < 0.001) compared to the “before COVID onset” patients. There were statistical trends for prior cerebral vascular accidents, steroid use, and other major psychiatric disorders. For complications arising during the hospital course, only stroke/cerebral vascular accident was statistically different between patient groups.

Table 5 summarizes patient hospital courses stratified by “before COVID onset” and “after COVID onset” patients. The day of the week patients were admitted (weekends vs. weekdays) remained similar between the two groups. Important to the primary objective of this retrospective analysis, there were no statistical differences in the percentage of patients receiving PT nor the median days to mobilization in the ICU between the before and “after COVID onset” groups (Table 5). Approximately 65% of patients received a PT order and the median time to mobilization was 4 days “before COVID onset” and 3 days “after COVID onset” (*p* = 0.86). The lengths of stay in the hospital, in the ICU, and on a ventilator did not significantly differ before vs. “after COVID onset” (Figure 3D). In agreement with “after COVID onset” patients having a greater injury severity, lower predicted survivability, and greater total complications, there was also greater mortality associated with the “after COVID onset” patients, going from 4.8% mortality in the “before COVID onset” group to 10.7% mortality in the “after COVID onset” group (*p* = 0.001). “after COVID onset” patients were also more likely to be readmitted (14% vs. 6%, *p* < 0.001) compared to “before COVID onset” patients.

### 3.2. “After COVID Onset” Patient Characteristics, Injury Attributes, and Outcomes Stratified by Mobilization

Our secondary analysis examined patient attributes and hospital courses within the “after COVID onset” group based on whether or not patients were mobilized. Of the 499 “after COVID onset” patients, those that were mobilized were older (median age: 57 vs. 50 years, *p* = 0.005), female (74% female Mobilized vs. 68% male Mobilized, *p* = 0.004), presented with a blunt injury, and had not sustained a gunshot wound (Table 6). In fact, patients who sustained a gunshot wound were 55% less likely to be mobilized compared to patients classified with chief complaints of falls and motor vehicle crashes. There were no statistical differences between patient groups for basic clinical outcomes (Table 6).

**Figure 4.**
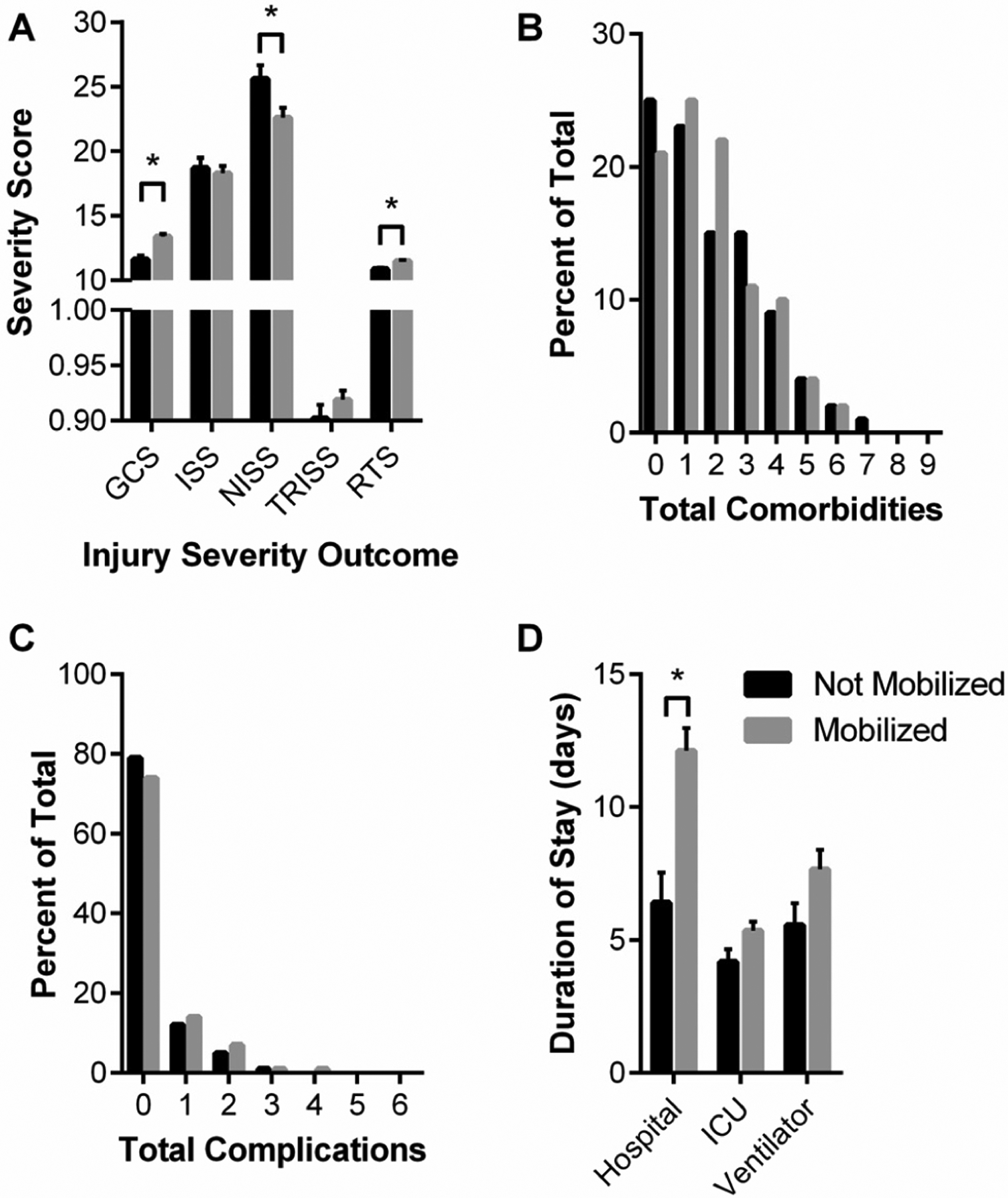
“after COVID onset” Mobilized and Not Mobilized patient injury severity (**A**), cumulative complications (**B**), cumulative comorbidities (**C**), and hospital durations (**D**). (**A**) Glasgow Coma Score (GCS), Injury Severity Score (ISS), New Injury Severity Score (NISS), Trauma Score and Injury Severity Score (TRISS), and Revised Trauma Score (RTS) are shown as median and interquartile range. Data analyzed using Mann–Whitney test with * representing statistical difference between groups (*p* < 0.05). (**B**) Chi-squared analysis of distribution shifts of total patient complications. (**C**) Chisquared analysis of distribution shifts of total patient comorbidities. (**D**) Hospital, ICU, and ventilator durations are shown as median and interquartile range. Data analyzed using Mann–Whitney test with * representing statistical difference between groups (*p* < 0.05).

In regard to patient injury severity, the proportion of mobilized patients classified as severely injured was not statistically different from the proportion of patients not mobilized (~58%); however, mobilized patients had greater GCS and RTS scores and lower NISS scores (Figure 4A). GCS is a predictive coma score such that a “15” is normal and lower scores are associated with a greater likelihood of coma, vegetative state, and ultimately death. The RTS is a trauma score that correlates statistically with greater survival. There were no significant differences between patient groups for total comorbidities presented in the ICU nor total complications experienced within the ICU (Figure 4B,C).

Individual comorbidities and complications for the Mobilized vs. Not Mobilized groups are reported in Tables 7 and 8, respectively. A lesser percentage of Mobilized patients presented to the ICU with advance directives limiting care (~6% vs. 24%, *p* < 0.001) compared to the Not Mobilized patients. There were statistical trends for hypertension and cirrhosis. For complications arising during the hospital course of care, there were only statistical trends for myocardial infarctions and pulmonary embolism.

Table 9 summarizes patient hospital courses stratified by “after COVID onset” patient mobilization. The day of the week and weekday vs. weekend were not associated with whether or not a patient was mobilized. Interestingly, with respect to patient outcomes, patients who were mobilized were more likely to be discharged alive (98% vs. 72%, *p* < 0.001), although they are also more likely to be readmitted (18% vs. 6%, *p* < 0.001). Mobilized patients’ median hospital stay was greater (8 vs. 3 days, *p* < 0.001), but ICU duration and days on a ventilator were not different between the groups (Figure 4D).

## 4. Discussion

Our retrospective study revealed that there was an association between the onset of COVID-19 and ICU patients with greater injury severity, complications, comorbidities, and mortality rate. There was also evidence that the types of injury patients presented to the ICU shifted after the onset of COVID-19, in agreement with other studies [27,28]. Despite these changes, we report herein that there was no difference in the proportion of patients receiving a PT consult or time to mobilization for patient groups “before COVID onset” vs. “after COVID onset”. Patient injury characteristics notwithstanding, this is a significant finding considering the early uncertainty of the communicable COVID-19 disease.

In the two years following the start of the COVID-19 pandemic, the most significant change seen among the different groups was a rise in the severity of trauma injuries requiring patient admission to the ICU. The consensus among the literature suggests the main cause of these findings to be a delay in patients seeking care due to fear of COVID-19 infection [29,30]. Specifically, in Switzerland, Burgard et al. found a significant decrease in acute appendicitis consults but a significant increase in cases of complicated appendicitis, indicating that patients were likely waiting for a longer period of time before presenting to the ED [31]. Herein, the proportion of patients classified as “severely injured” was greater in the “after COVID onset” group, and additionally, we detected total comorbidity shifts, suggesting patients were delaying their care to avoid any healthcare setting. Of the twenty-three comorbidities we tracked, it is notable that the number of patients presenting with disseminated cancer was greater in the “after COVID onset” group (Table 3). We ran ANCOVAs to control for either cumulative patient complications or disseminated cancer, and there was still no significant difference between “before COVID onset” and “after COVID onset” groups in days to mobilization (*p* ≥ 0.779). This retrospective analysis suggests that the PT staff at this level II trauma center were able to manage the shift in patient injury attributes and complications. The extent to which these results would be similar to a level I trauma center are unclear.

Recent studies have attempted to capture changes in patient characteristics of trauma ICU admissions after the COVID-19 pandemic forced adjustment to the lives of populations around the globe [32–35]. In a large, multicenter retrospective study that analyzed the first three months of the stay-at-home order, Yeates et al. found that “after COVID onset” trauma patients had decreased lengths of stay in both ICU and total hospital days [32]. These findings are mirrored among other studies that looked at the changes in admission characteristics in the time period immediately following the installment of the stay-at-home orders [36–38]. The results of this study show that there were no major changes in patient demographics; however, the types of injuries resulting in ICU admittance did shift. There was no change in the percentage of penetrating trauma compared to blunt trauma, but other studies, including one conducted in south London, found a decrease in blunt trauma presentations for oral and maxillofacial trauma consults [39]. There was a greater percentage of gunshot wounds as a chief complaint, which is similar to findings reported by others [27,28]. However, Pettke et al. found that in South Africa, assaults decreased during the strictest lockdown period, which is in contrast with our findings, reflecting a difference in patient populations [40].

In this study, we also found that the percentage of patients who were discharged in an expired status increased significantly after the beginning of the COVID-19 pandemic, which reflects findings at a tertiary hospital trauma center in China, supporting a worldwide increase in mortality due to the pandemic [41]. However, this study found that the severity of trauma decreased after the beginning of the COVID-19 pandemic, which is consistent with the theory that fewer people out of their homes leads to fewer severe injuries, especially secondary to etiologies such as motor vehicle collisions. At a tertiary hospital in China, a similar decrease in trauma severity was observed, and in Greece, a decrease in patient presentation due to motor vehicle accidents was observed [42].

This study also sought to investigate the relationship between the COVID-19 pandemic and physical therapy consultation. Our analysis of “after COVID onset” patients exclusively who did or did not receive mobilization revealed that mobilized patients had a greater hospital duration compared to those who were not mobilized. Previous experimental studies found early mobility in the ICU to be associated with fewer days on mechanical ventilation [21] and fewer days spent both in the ICU and in the hospital [4–13,43]. It is possible that the effectiveness of the mobilization protocol differed between this retrospective study and the experimental studies where the protocol’s implementation could be tightly controlled. It is also possible that the retrospective vs. experimental studies’ differences in outcomes could be due to differing patient characteristics such as injury severity, case management decisions such as discharge disposition, differences in physician decisions regarding extubation, or perhaps the level of mobilization attained by the patients.

In the last few decades, viral pandemics that have had global effects have been met with major strategies to combat the spread among the population. The COVID-19 pandemic has been the most recent and has had the most significant response on a global scale involving major social, healthcare, and international changes [44]. Whether or not these changes to the characteristics of patient populations are permanent remains to be elucidated. Research on the H1N1 influenza pandemic of 2009, which was the most recent prior to the SARS-CoV-2 pandemic, describes various impacts on healthcare institutions including overwhelmed EDs and changes to staffing to overcome pandemic surges that have highlighted the need for institutional preparedness [45]. Health institutional changes from the lessons learned during the previous pandemics and during the time period immediately following the start of the COVID-19 pandemic including changes to length of stay, time to transfer, and time to intervention for non-COVID trauma patients; early medical interventions including antivirals; and supportive therapies to alleviate milder symptoms. As such, hospitals and healthcare institutions may require a shift in expectations of potential trauma admissions up to two years after a global pandemic. This may involve the formation of guidelines in addition to pandemic preparedness for the reallocation of resources to adjust for a switch in more severely injured patients during and in the years after a pandemic including availability of PT, further training of personnel, and hospital resources to meet the needs of more severely injured trauma patients.

The limitations of this study include its retrospective nature that relies on accurate documentation and could be influenced by charting discrepancies. Another limitation is the lack of analysis of further details that were not collected for each patient’s mobilization sessions, such as frequency of sessions, average duration of sessions, and the types of mobilization. Additionally, retrospective analysis cannot determine the causation of the reported findings and can only evaluate associations or relationships among the patient characteristics and outcomes. The focus on cases from a single institution may lack external validity. As such, future studies should focus on the long-term effects of COVID-19 on regional and national levels. Furthermore, the limitations of the hospital in this study, including its level II status, potentially involve the transfer of more severe injuries to outside hospitals, which may skew the reproducibility among hospitals of different capacities and patient populations.

## Figures and Tables

**Table 1.** Injury Severity Score definition and predictive utility.

Severity Scoring System	Determining Variables	Utility
Glasgow Coma Scale (GCS) Scale 3 (high severity) to 15 (low severity)	Eye response, verbal response, motor response	Higher scores correlate with better neurological function
Revised Trauma Score (RTS) Scale 0 (high severity) to 12 (low severity)	GCS, systolic pressure, respiratory rate	Higher scores correlate with greater survival rate and better outcome
Injury Severity Score (ISS) Scale 1 (low severity) to 75 (high severity)	Top 3 body region injuries	Higher scores correlate with low probability of survival
New Injury Severity Score (NISS) Scale 1 (low severity) to 75 (high severity)	Top 3 injuries regardless of body region	Higher scores correlate with low probability of survival
Trauma and Injury Severity Score (TRISS) Scale 0 (high severity) to 1 (low severity)	RTS and ISS	Higher scores correlate with high probability of survival

**Table 2.** Patient characteristics stratified by before and after COVID onset.

Patient Characteristics	Before COVID Onset (*n* = 378)	After COVID Onset (*n* = 499)	*p* Value
Age at Admission, years (IQR)	50 (42.25)	55 (40)	0.70
Gender, *n* (%)			0.86
Male	244 (64.6%)	325 (65.1%)	
Female	134 (35.4%)	174 (34.9%)	
Ethnicity, *n* (%)			0.46
Hispanic or Latino	16 (4.0%)	24 (4.8%)	
Not Hispanic or Latino	361 (96.0%)	475 (95.2%)	
Race, *n* (%)			0.016
White or Caucasian	305 (80.7%)	368 (73.8%)	
Other (Asian, Black/African American, Native Hawaiian, Pacific Islander, Refused)	73 (19.3%)	131 (26.2%)	
Vitals			
Height, meters (IQR)	1.73 (0.15)	1.73 (0.15)	1.73 (0.15)
Weight, kilograms (IQR)	79.9 (27.03)	80.45 (29.88)	80.45 (29.88)
Body Mass Index (IQR)	26.89 (8.58)	26.65 (8.40)	26.65 (8.40)
Systolic Blood Pressure (IQR)	132 (36.25)	132 (34)	132 (34)
Diastolic Blood Pressure (IQR)	81 (22.25)	81 (22)	81 (22)
Pulse (IQR)	89 (29)	87 (30)	87 (30)
Respiratory Rate (IQR)	18 (4)	18 (6)	18 (6)
SpO_2_ (IQR)	97 (5)	97 (4)	97 (4)
Injury type, *n* (%)			0.29
Blunt	349 (92.3%)	447 (89.6%)	
Penetrating	29 (7.7%)	51 (10.2%)	
Other (thermal, not applicable)	0	1 (0.2%)	
Chief mechanisms of injury, *n* (%)			0.046
Fall	161 (42.6%)	176 (35.3%)	
Gunshot wound	17 (4.5%)	41 (8.2%)	
Motor vehicle crash	177 (46.8%)	250 (50.1%)	
Other (assault, bicycle, burn, knife, pedestrian, puncture wound, other blunt mechanism, other penetrating mechanism, struck by object or person, unknown)	23 (6.1%)	32 (6.4%)	
Injury severity			
Severe ISS, *n* (%)	177 (46.83%)	292 (58.52%)	0.001
TRISS (IQR)	0.968 (0.044)	0.965 (0.068)	0.014
ISS (IQR)	14 (13)	17 (15)	<0.001
NISS (IQR)	17 (16)	22 (16)	<0.001
RTS (IQR)	12 (1)	12 (1)	0.21

IQR = interquartile range.

**Table 3.** Comorbidities by before and after COVID onset.

Comorbidities (%)	Before COVID Onset (*n* = 378)	After COVID Onset (*n* = 499)	*p* Value
Total comorbidities (IQR)	1 (2.25)	2 (2)	0.0067
Currently receiving chemotherapy	2 (0.53%)	5 (1.00%)	0.44
Congenital anomalies	3 (0.79%)	2 (0.40%)	0.44
Congestive heart failure	29 (7.67%)	52 (10.42%)	0.16
Smoker	91 (24.07%)	136 (27.25%)	0.29
Renal failure	29 (7.67%)	45 (9.02%)	0.48
Prior cerebral vascular accident	23 (6.08%)	48 (9.62%)	0.057
Diabetes	77 (20.37%)	83 (16.63%)	0.16
Disseminated cancer	2 (0.53%)	30 (6.01%)	<0.0001
Advanced directive limiting care	8 (2.12%)	63 (12.63%)	<0.0001
History of angina	1 (0.26%)	1 (0.20%)	0.84
Prior myocardial infarction	16 (4.23%)	31 (6.21%)	0.20
Peripheral vascular disease	19 (5.03%)	28 (5.61%)	0.70
Hypertension	161 (42.59%)	204 (40.88%)	0.61
COPD	30 (7.94%)	35 (7.01%)	0.61
Steroid use	0 (0%)	5 (1.00%)	0.051
Cirrhosis	9 (2.38%)	12 (2.40%)	0.98
Other major non-psychiatric disorder	1 (0.27%)	4 (0.8%)	0.30
EtOH use disorder	37 (9.79%)	59 (11.82%)	0.34
Other substance use disorder	41 (10.85%)	68 (13.63%)	0.22
Dementia	19 (5.03%)	18 (3.61%)	0.30
ADHD	10 (2.65%)	9 (1.80%)	0.40
Other major psychiatric disorder	65 (17.20%)	112 (22.44%)	0.0551

**Table 4.** Complications before and after COVID onset.

Complications (%)	Before COVID Onset (*n* = 378)	After COVID Onset (*n* = 499)	*p* Value
Total complications (IQR)	0 (0)	0 (1)	0.0046
Deep surgical infection	4 (1.06%)	5 (1.00%)	0.93
Drug/EtOH substance use disorder	4 (1.06%)	8 (1.06%)	0.49
Deep venous thrombosis (DVT)	15 (3.97%)	21 (4.21%)	0.86
Compartment syndrome	1 (0.26%)	2 (0.40%)	0.73
Graft/prosthesis/flap failure	0 (0%)	1 (0.20%)	0.38
Myocardial infarction	0 (0%)	2 (0.40%)	0.22
Organ space infection	0 (0%)	2 (0.40%)	0.22
Osteomyelitis	4 (1.06%)	1 (0.20%)	0.095
Pneumonia	43 (11.38%)	58 (11.62%)	0.91
Pulmonary embolism	3 (0.79%)	7 (1.40%)	0.40
Sepsis	16 (4.23%)	23 (6.81%)	0.10
Stroke/cerebral vascular accident	2 (0.53%)	12 (2.40%)	0.028
Superficial infection	3 (0.79%)	5 (1.00%)	0.75
Urinary tract infection	11 (2.91%)	14 (2.81%)	0.93

**Table 5.** Hospital stay characteristics stratified by before and after COVID onset.

Hospital Stay Characteristics	Before COVID Onset (*n* = 378)	After COVID Onset (*n* = 499)	*p* Value
Weekend vs. Weekday Admission, *n* (%)			0.66
Weekday	263 (69.6%)	354 (70.9%)	
Weekend	115 (30.4%)	145 (29.1%)	
Day of the Week Admission, *n* (%)			0.51
Sunday	48 (12.7%)	71 (14.2%)	
Monday	57 (15.1%)	71 (14.2%)	
Tuesday	53 (14.0%)	58 (11.6%)	
Wednesday	42 (11.1%)	75 (15.0%)	
Thursday	56 (14.8%)	72 (14.4%)	
Friday	55 (14.6%)	78 (15.6%)	
Saturday	67 (17.7%)	74 (14.8%)	
Received PT, *n* (%)	215 (56.9%)	302 (60.6%)	0.97
PT Time to Mobilization, day (IQR)	4 (4)	3 (3.25)	0.86
Discharge Status, *n* (%)			0.001
Alive	360 (95.2%)	445 (89.2%)	
Expired	18 (4.8%)	54 (10.8%)	
Readmission			
Patients Readmitted, *n* (%)	23 (6.08%)	70 (14.03%)	0.0003
Days Post ED Before Readmission, days (IQR)	23.92 (37.47)	42.00 (83.21)	0.04
Length of Readmission Stay, days (IQR)	5 (10)	2 (3.92)	0.03

IQR = interquartile range.

**Table 6.** Patient characteristics after COVID stratified by mobilization.

Patient Characteristics	Not Mobilized (*n* = 171)	Mobilized (*n* = 328)	*p* Value
Age at Admission, years (IQR)	50 (38)	57 (41)	0.005
Gender, *n* (%)			0.004
Male	126 (73.7%)	199 (60.7%)	
Female	45 (26.3%)	129 (39.3%)	
Ethnicity, *n* (%)			0.57
Hispanic or Latino	6 (3.5%)	13 (4.0%)	
Not Hispanic or Latino	164 (96.5%)	309 (96.0%)	
Race, *n* (%)			0.51
White or Caucasian	123 (71.9%)	245 (74.7%)	
Other (Asian, Black/African American, Native Hawaiian, Pacific Islander, Refused)	48 (28.1%)	83 (25.3%)	
Vitals			
Height, meters (IQR)	1.75 (0.15)	1.727 (0.17)	0.071
Weight, kilograms (IQR)	81.6 (29.7)	79.5 (30.7)	0.95
Body Mass Index (IQR)	26.75 (8.81)	26.63 (8.32)	0.57
Systolic Blood Pressure (IQR)	131 (47)	132.5 (32.49)	0.78
Diastolic Blood Pressure (IQR)	81 (24)	81 (21.75)	0.091
Pulse (IQR)	88 (27)	86.5 (32)	0.25
Respiratory Rate (IQR)	18 (6)	18 (6)	0.79
SpO_2_ (IQR)	98 (5)	97 (4)	0.33
Injury Type, *n* (%)			<0.001
Blunt	141 (82.4%)	305 (93.3%)	
Penetrating	30 (17.5%)	21 (6.4%)	
Other (thermal, not applicable)	0 (0%)	1 (0.3%)	
Chief Mechanisms of Injury, *n* (%)			<0.001
Fall	53 (30.1%)	123 (37.5%)	
Gunshot wound	26 (15.2%)	15 (4.6%)	
Motor vehicle crash	76 (44.4%)	174 (53.0%)	
Other (assault, bicycle, burn, knife, pedestrian, puncture wound, other blunt mechanism, other penetrating mechanism, struck by object or person, unknown)	16 (9.4%)	16 (4.9%)	
Injury Severity			
Severe ISS, *n* (%)	99 (33.9%)	193 (66.1%)	<0.001
TRISS (IQR)	0.97 (0.0985)	0.97 (0.056)	0.27
ISS (IQR)	17 (16)	17 (14)	0.73
NISS (IQR)	22 (22)	22 (15)	0.028
RTS (IQR)	12 (2)	2 (0.75)	<0.001

IQR = interquartile range.

**Table 7.** Comorbidities after COVID stratified by mobilization.

Comorbidities (%)	Not MobiHzed (*n* = 171)	Mobilized (*n* = 328)	*p* Value
Total comorbidities (IQR)	2 (3)	2 (2)	0.089
Currently receiving chemotherapy	3 (1.75%)	2 (0.61%)	0.22
Congenital anomalies	1 (0.58%)	1 (0.30%)	0.64
Congestive heart failure	18 (10.53%)	34 (10.37%)	0.96
Smoker	53 (30.99%)	83 (25.30%)	0.18
Renal failure	15 (8.77%)	30 (9.15%)	0.89
Prior cerebral vascular accident	17 (9.94%)	31 (9.45%)	0.86
Diabetes	32 (18.71%)	51 (15.55%)	0.37
Disseminated cancer	12 (7.02%)	18 (5.49%)	0.50
Advanced directive limiting care	42 (24.56%)	21 (6.40%)	<0.0001
History of angina	0 (0%)	1 (0.30%)	0.47
Prior myocardial injarctton	10 (5.85%)	21 (6.40%)	0.81
Peripheral vascular disease	11 (6.43%)	17 (5.18%)	0.56
Hypertension	61 (35.65%)	143 (43.60%)	0.087
COPD	11 (6.43%)	24 (7.32%)	0.71
Steroid use	0 (0%)	5 (1.52%)	0.10
Cirrhosis	8 (4.68%)	4 (1.22%)	0.017
Other major non-psychiatric disorder	2 (1.17%)	2 (0.61%)	0.51
EtOH use disorder	25 (14.62%)	34 (10.37%)	0.16
Other substance use disorder	29 (16.96%)	39 (11.89%)	0.12
Dementia	7 (4.09%)	11 (3.35%)	0.67
ADHD	3 (1.75%)	6 (1.83%)	0.95
Other major psychiatric disorder	33 (19.30%)	79 (24.09%)	0.22

**Table 8.** Complications after COVID stratified by mobilization.

Complications (%)	Not Mobilized (*n* = 171)	Mobilized (*n* = 328)	*p* Value
Total complications (IQR)	0 (1)	0 (1)	0.5775
Deep surgical infection	0 (0%)	5 (1.52%)	0.10
Drug/EtOH substance use disorder	2 (1.17%)	6 (1.83%)	0.58
Deep venous thrombosis (DVT)	8 (4.68%)	13 (3.96%)	0.71
Compartment syndrome	0 (0%)	2 (0.61%)	0.31
Graft/prosthesis/flap failure	0 (0%)	1 (0.30%)	0.47
Myocardial infarction	2 (1.17%)	0 (0%)	0.05
Organ space infection	0 (0%)	2 (0.61%)	0.31
Osteomyelitis	0 (0%)	1 (0.30%)	0.47
Pneumonia	20 (11.70%)	38 (11.59%)	0.97
Pulmonary embolism	0 (0%)	7 (2.13%)	0.054
Sepsis	11 (6.43%)	23 (7.01%)	0.81
Stroke/cerebral vascular accident	6 (3.51%)	6 (1.83%)	0.25
Superficial infection	0 (0%)	5 (1.52%)	0.10
Urinary tract infection	2 (1.17%)	12 (3.66%)	0.11

**Table 9.** Hospital stay characteristics after COVID stratified by PT and no PT.

Hospital Stay Characteristics	Not Mobilized (*n* = 171)	Mobilized (*n* = 328)	*p* Value
Weekend vs. Weekday Admission, *n* (%)			0.785
Weekday	120 (70.2%)	234 (71.3%)	
Weekend	51 (29.8%)	94 (28.6%)	
Day of the Week Admission, *n* (%)			0.292
Sunday	20 (11.7%)	51 (15.5%)	
Monday	25 (14.6%)	46 (14.0%)	
Tuesday	18 (10.5%)	40 (12.2%)	
Wednesday	21 (12.3%)	54 (16.5%)	
Thursday	31 (18.1%)	41 (12.5%)	
Friday	25 (14.6%)	53 (16.2%)	
Saturday	31 (18.1%)	43 (13.1%)	
PT Time to Mobilization, day (IQR)	-	3 (3.25)	-
Hospital Days, day (IQR)	3 (4)	8 (8)	<0.001
Intensive Care Unit Days, day (IQR)	2 (2)	3 (3)	0.053
Ventilatory Days, day (IQR)	3 (4)	4 (8)	0.076
Total Complications, *n* (IQR)	0 (1)	0 (1)	0.578
Discharge Status, *n* (%)			<0.001
Alive	124 (72.5%)	321 (97.9%)	
Expired	47 (27.5%)	7 (2.1%)	
Readmission			
Readmission			0.001
No Patient Readmission, *n* (%)	161 (94.2%)	268 (81.7%)	
Patients Readmission, *n* (%)	10 (5.8%)	60 (18.3%)	
Days Post ED Before Readmission, days (IQR)	68.76 (91.05)	34.68 (87.52)	0.908
Length of Readmission Stay, days (IQR)	2 (4.69)	3 (3.46)	0.781

IQR = interquartile range.

## Data Availability

The datasets presented in this article are not readily available due to hospital patient privacy policies. Requests to access the datasets should be directed to the corresponding author.

## References

[1] Talic S; Shah S; Wild H; Gasevic D; Maharaj A; Ademi Z; Li X; Xu W; Mesa-Eguiagaray I; Rostron J; Effectiveness of public health measures in reducing the incidence of COVID-19, SARS-CoV-2 transmission, and COVID-19 mortality: Systematic review and meta-analysis. BMJ 2021, 375, e068302. 34789505 10.1136/bmj-2021-068302 PMC9423125

[2] Birkmeyer JD; Barnato A; Birkmeyer N; Bessler R; Skinner J The Impact of the COVID-19 Pandemic on Hospital Admissions in the United States. Health Aff. 2020, 39, 2010–2017. 10.1377/hlthaff.2020.00980 PMC7769002 32970495

[3] Hafner K Fear of COVID-19 leads other patients to decline critical treatment. The New York Times, 25 May 2020.

[4] Azuh O; Gammon H; Burmeister C; Frega D; Nerenz D; DiGiovine B; Siddiqui A Benefits of Early Active Mobility in the Medical Intensive Care Unit: A Pilot Study. Am. J. Med 2016, 129, 866–871.e861. 27107920 10.1016/j.amjmed.2016.03.032

[5] Corcoran JR; Herbsman JM; Bushnik T; Van Lew S; Stolfi A; Parkin K; McKenzie A; Hall GW; Joseph W; Whiteson J; Early Rehabilitation in the Medical and Surgical Intensive Care Units for Patients with and without Mechanical Ventilation: An Interprofessional Performance Improvement Project. PM R 2017, 9, 113–119. 27346093 10.1016/j.pmrj.2016.06.015

[6] Deng H; Chen J; Li F; Li-Tsang CW; Liu Q; Ma X; Ao M; Chen N; Zhou Y; Zhong X; Effects of mobility training on severe burn patients in the BICU: A retrospective cohort study. Burns 2016, 42, 1404–1412. 27595451 10.1016/j.burns.2016.07.029

[7] Engel HJ; Tatebe S; Alonzo PB; Mustille RL; Rivera MJ Physical therapist-established intensive care unit early mobilization program: Quality improvement project for critical care at the University of California San Francisco Medical Center. Phys. Ther 2013, 93, 975–985. 23559525 10.2522/ptj.20110420

[8] Engel HJ; Needham DM; Morris PE; Gropper MA ICU early mobilization: From recommendation to implementation at three medical centers. Crit. Care Med 2013, 41, S69–S80. 23989097 10.1097/CCM.0b013e3182a240d5

[9] Lai CC; Chou W; Chan KS; Cheng KC; Yuan KS; Chao CM; Chen CM Early Mobilization Reduces Duration of Mechanical Ventilation and Intensive Care Unit Stay in Patients with Acute Respiratory Failure. Arch. Phys. Med. Rehabil 2017, 98, 931–939. 27979608 10.1016/j.apmr.2016.11.007

[10] McWilliams D; Weblin J; Atkins G; Bion J; Williams J; Elliott C; Whitehouse T; Snelson C Enhancing rehabilitation of mechanically ventilated patients in the intensive care unit: A quality improvement project. J. Crit. Care 2015, 30, 13–18. 25316527 10.1016/j.jcrc.2014.09.018

[11] Morris PE; Goad A; Thompson C; Taylor K; Harry B; Passmore L; Ross A; Anderson L; Baker S; Sanchez M; Early intensive care unit mobility therapy in the treatment of acute respiratory failure. Crit. Care Med 2008, 36, 2238–2243. 18596631 10.1097/CCM.0b013e318180b90e

[12] Schaller SJ; Anstey M; Blobner M; Edrich T; Grabitz SD; Gradwohl-Matis I; Heim M; Houle T; Kurth T; Latronico N; Early, goal-directed mobilisation in the surgical intensive care unit: A randomised controlled trial. Lancet 2016, 388, 1377–1388. 27707496 10.1016/S0140-6736(16)31637-3

[13] Sigler M; Nugent K; Alalawi R; Selvan K; Tseng J; Edriss H; Turner A; Valdez K; Krause D Making of a Successful Early Mobilization Program for a Medical Intensive Care Unit. South Med. J 2016, 109, 342–345. 27255089 10.14423/SMJ.0000000000000472

[14] Hanekom SD; Louw Q; Coetzee A The way in which a physiotherapy service is structured can improve patient outcome from a surgical intensive care: A controlled clinical trial. Crit. Care 2012, 16, R230. 23232109 10.1186/cc11894 PMC3672619

[15] Klein K; Mulkey M; Bena JF; Albert NM Clinical and psychological effects of early mobilization in patients treated in a neurologic ICU: A comparative study. Crit. Care Med 2015, 43, 865–873. 25517476 10.1097/CCM.0000000000000787

[16] Pandullo SM; Spilman SK; Smith JA; Kingery LK; Pille SM; Rondinelli RD; Sahr SM Time for critically ill patients to regain mobility after early mobilization in the intensive care unit and transition to a general inpatient floor. J. Crit. Care 2015, 30, 1238–1242. 26346813 10.1016/j.jcrc.2015.08.007

[17] Titsworth WL; Hester J; Correia T; Reed R; Guin P; Archibald L; Layon AJ; Mocco J The effect of increased mobility on morbidity in the neurointensive care unit. J. Neurosurg 2012, 116, 1379–1388. 22462507 10.3171/2012.2.JNS111881

[18] Adler J; Malone D Early mobilization in the intensive care unit: A systematic review. Cardiopulm. Phys. Ther. J 2012, 23, 5–13. PMC3286494 22807649

[19] Bailey P; Thomsen GE; Spuhler VJ; Blair R; Jewkes J; Bezdjian L; Veale K; Rodriquez L; Hopkins RO Early activity is feasible and safe in respiratory failure patients. Crit. Care Med 2007, 35, 139–145. 17133183 10.1097/01.CCM.0000251130.69568.87

[20] Burtin C; Clerckx B; Robbeets C; Ferdinande P; Langer D; Troosters T; Hermans G; Decramer M; Gosselink R Early exercise in critically ill patients enhances short-term functional recovery. Crit. Care Med 2009, 37, 2499–2505. 19623052 10.1097/CCM.0b013e3181a38937

[21] Hodgson CL; Bailey M; Bellomo R; Berney S; Buhr H; Denehy L; Gabbe B; Harrold M; Higgins A; Iwashyna TJ; A Binational Multicenter Pilot Feasibility Randomized Controlled Trial of Early Goal-Directed Mobilization in the ICU. Crit. Care Med 2016, 44, 1145–1152. 26968024 10.1097/CCM.0000000000001643

[22] Laurent H; Aubreton S; Richard R; Gorce Y; Caron E; Vallat A; Davin AM; Constantin JM; Coudeyre E Systematic review of early exercise in intensive care: A qualitative approach. Anaesth. Crit. Care Pain Med 2016, 35, 133–149. 26655865 10.1016/j.accpm.2015.06.014

[23] Needham DM; Korupolu R; Zanni JM; Pradhan P; Colantuoni E; Palmer JB; Brower RG; Fan E Early physical medicine and rehabilitation for patients with acute respiratory failure: A quality improvement project. Arch. Phys. Med. Rehabil 2010, 91, 536–542. 20382284 10.1016/j.apmr.2010.01.002

[24] Patman SM; Dennis DM; Hill K Exploring the capacity to ambulate after a period of prolonged mechanical ventilation. J. Crit. Care 2012, 27, 542–548. 22421005 10.1016/j.jcrc.2011.12.020

[25] Schweickert WD; Pohlman MC; Pohlman AS; Nigos C; Pawlik AJ; Esbrook CL; Spears L; Miller M; Franczyk M; Deprizio D; Early physical and occupational therapy in mechanically ventilated, critically ill patients: A randomised controlled trial. Lancet 2009, 373, 1874–1882. 19446324 10.1016/S0140-6736(09)60658-9 PMC9906655

[26] AOTR. AOTR Trauma Registry Resource Manual; AOTR: Columbus, OH, USA, 2020; p. 48.

[27] Strassle PD; Ko JS; Ponder M; Napoles AM; Kinlaw AC; Schiro SE Impact of COVID-related policies on gunshot wound assault hospitalizations in the United States: A statewide time series analysis. Inj. Epidemiol 2023, 10, 2. 36624533 10.1186/s40621-022-00412-7 PMC9829223

[28] Padubidri AA; Rushing A; Ochenjele G; Sontich J; Napora J; Osborne A; Delozier S; Wetzel R Increase in gunshot wounds at a level 1 trauma center following the COVID19 pandemic. OTA Int. 2021, 4, e159. 34805774 10.1097/OI9.0000000000000159 PMC8598221

[29] Hartnett KP; Kite-Powell A; DeVies J; Coletta MA; Boehmer TK; Adjemian J; Gundlapalli AV; National Syndromic Surveillance Program Community of Practice. Impact of the COVID-19 Pandemic on Emergency Department Visits—United States, 1 January 2019–30 May 2020. MMWR Morb. Mortal. Wkly. Rep 2020, 69, 699–704. 32525856 10.15585/mmwr.mm6923e1 PMC7315789

[30] Oseran AS; Nash D; Kim C; Moisuk S; Lai PY; Pyhtila J; Sequist TD; Wasfy JH Changes in hospital admissions for urgent conditions during COVID-19 pandemic. Am. J. Manag. Care 2020, 26, 327–328. 32835458 10.37765/ajmc.2020.43837

[31] Burgard M; Cherbanyk F; Nassiopoulos K; Malekzadeh S; Pugin F; Egger B An effect of the COVID-19 pandemic: Significantly more complicated appendicitis due to delayed presentation of patients! PLoS ONE 2021, 16, e0249171. 34032800 10.1371/journal.pone.0249171 PMC8148360

[32] Yeates EO; Grigorian A; Schellenberg M; Owattanapanich N; Barmparas G; Margulies D; Juillard C; Garber K; Cryer H; Tillou A; Decreased hospital length of stay and intensive care unit admissions for non-COVID blunt trauma patients during the COVID-19 pandemic. Am. J. Surg 2022, 224, 90–95. 35219493 10.1016/j.amjsurg.2022.02.055 PMC8863305

[33] Huang GS; Chance EA; Dunham CM Influence of a Stay-At-Home Order on Trauma Volume and Injury Patterns at a Level I Trauma Center in Ohio. Am. Surg 2021, 89, 31348211047488. 10.1177/00031348211047488 34732068

[34] Bologheanu R; Maleczek M; Laxar D; Kimberger O Outcomes of non-COVID-19 critically ill patients during the COVID-19 pandemic: A retrospective propensity score-matched analysis. Wien. Klin. Wochenschr 2021, 133, 942–950. 33909109 10.1007/s00508-021-01857-4 PMC8080479

[35] Park J; Jung K; Kwon J; Moon J; Huh Y; Heo YJ; Kang BH Changes in the characteristics of trauma patients after the early COVID-19 outbreak: A retrospective study of a regional level 1 trauma center in Republic of Korea. Medicine 2022, 101, e28567. 35029226 10.1097/MD.0000000000028567 PMC8758017

[36] Maman Y; Lee Goldstein A; Neeman U; Lessing Y; Orbach L; Sirhan S; Falk E; Lahat G The Impact of the COVID-19 Pandemic on an Israeli Acute Care Surgery Unit: Fewer Patients, More Disease. Am. Surg 2022, 88, 2863–2870. 33856956 10.1177/00031348211011132 PMC9629022

[37] Leichtle SW; Rodas EB; Procter L; Bennett J; Schrader R; Aboutanos MB The influence of a statewide “Stay-at-Home” order on trauma volume and patterns at a level 1 trauma center in the united states. Injury 2020, 51, 2437–2441. 32798035 10.1016/j.injury.2020.08.014 PMC7414300

[38] Dayananda KSS; Mercer ST; Agarwal R; Yasin T; Trickett RW A comparative review of 1,004 orthopaedic trauma patients before and during the COVID-19 pandemic. Bone Jt. Open 2020, 1, 568–575. 33215147 10.1302/2633-1462.19.BJO-2020-0121.R1 PMC7659692

[39] Yeung E; Brandsma DS; Karst FW; Smith C; Fan KFM The influence of 2020 coronavirus lockdown on presentation of oral and maxillofacial trauma to a central London hospital. Br. J. Oral. Maxillofac. Surg 2021, 59, 102–105. 33208288 10.1016/j.bjoms.2020.08.065 PMC7435349

[40] Pettke A; Stassen W; Laflamme L; Wallis LA; Hasselberg M Changes in trauma-related emergency medical services during the COVID-19 lockdown in the Western Cape, South Africa. BMC Emerg. Med 2023, 23, 72. 37370047 10.1186/s12873-023-00840-8 PMC10304331

[41] Shen B; Chen B; Li K; Cheng W; Mofatteh M; Regenhardt RW; Wellington J; Liang Z; Tang Q; Chen J; The Impact of COVID-19 Pandemic Lockdown on Emergency Department Visits in a Tertiary Hospital. Risk Manag. Healthc Policy 2023, 16, 1309–1316. 37489232 10.2147/RMHP.S415704 PMC10363383

[42] Sekadakis M; Katrakazas C; Michelaraki E; Kehagia F; Yannis G Analysis of the impact of COVID-19 on collisions, fatalities and injuries using time series forecasting: The case of Greece. Accid. Anal. Prev 2021, 162, 106391. 34525414 10.1016/j.aap.2021.106391 PMC8426576

[43] Ronnebaum JA; Weir JP; Hilsabeck TA Earlier Mobilization Decreases the Length of Stay in the Intensive Care Unit. Acute Care Phys. Ther 2012, 3, 204–210.

[44] Roychoudhury S; Das A; Sengupta P; Dutta S; Roychoudhury S; Choudhury AP; Ahmed ABF; Bhattacharjee S; Slama P Viral Pandemics of the Last Four Decades: Pathophysiology, Health Impacts and Perspectives. Int. J. Environ. Res. Public Health 2020, 17, 9411. 33333995 10.3390/ijerph17249411 PMC7765415

[45] Rubinson L; Mutter R; Viboud C; Hupert N; Uyeki T; Creanga A; Finelli L; Iwashyna TJ; Carr B; Merchant R; Impact of the fall 2009 influenza A(H1N1)pdm09 pandemic on US hospitals. Med. Care 2013, 51, 259–265. 23295577 10.1097/MLR.0b013e31827da8ea PMC6669026

